# Special Issue “The Characterization and Application of Enzymes in Bioprocesses”

**DOI:** 10.3390/ijms27125218

**Published:** 2026-06-09

**Authors:** Loredana Marcolongo

**Affiliations:** Research Institute on Terrestrial Ecosystems (IRET), National Research Council of Italy (CNR), Via Pietro Castellino 111, 80131 Naples, Italy; loredana.marcolongo@cnr.it

Enzymes are highly efficient biological catalysts that accelerate biochemical reactions in animals, plants and microorganisms without being consumed in the process. The unique feature of these biocatalysts is that they act with a high degree of specificity and efficiency, typically converting a single type of substrate (or at most a range of similar types) into product molecules by reducing the activation energy required for the reaction [1]. Cellular metabolism synthesises macromolecules through sequential enzymatic reactions via various distinct metabolic pathways. Each pathway comprises several stages, with specialised enzymes catalysing individual transformations of substrates (S) into products. Enzymatic specificity arises from evolutionarily refined active sites that bind and orient specific substrates, enabling highly efficient catalysis with minimal errors. The absence or limitation of a specific enzyme halts the flow of the pathway, often proving fatal to the cell [2].

The term ‘enzyme’ was coined in 1877 by Wilhelm Kühne, describing the ability of yeast to hydrolyse starch into glucose, and it is derived from the Greek words en (meaning ‘within’) and zume (meaning ‘yeast’). Almost a century of further research was needed to confirm that enzymes are proteins. In recent years, it has been shown that certain chemical reactions can be carried out by RNA molecules, known as ribozymes [3]. Victor Henri was the first to formalise enzyme kinetics, proposing the reversible formation of an enzyme–substrate (E-S) complex prior to catalysis, with the maximum reaction rate denoted as V(max). This was in response to the need to describe enzymatic activity in standard units that could be measured, so that different enzymes could be compared based on their rate and ability to bind to their substrates. This conceptual framework forms the basis of modern enzymology, emphasising substrate specificity, catalytic efficiency, and the interdependence of metabolic pathways in cellular homeostasis [4].

Thanks to their exceptional properties, enzymes play a crucial role in numerous bioprocesses. This is a key sector of biotechnology which uses living organisms and their enzymes to obtain a variety of products. They are essential for industry; unlike traditional chemical catalysts, biocatalysts significantly accelerate reactions under several conditions, demonstrate excellent substrate specificity to minimise by-products, and improve product quality. Biocatalysts also remain unchanged after the reaction, minimising energy consumption and making them sustainable and environmentally friendly alternatives [5].

In recent decades, there has been a surge in enzyme research, driven by advances in genomics, protein engineering, and synthetic biology. Enzymes are versatile and are used in many sectors (Figure 1), including agro-industry, detergents and textiles, medicine, pharmaceuticals, diagnostics and analytical devices, and the environmental sector for the production of biofuels, biomaterials, and bioremediation [6]. However, practical applications require ‘enhanced’ enzymes that are more stable at high temperatures, tolerant of organic solvents, and scalable to industrial volumes, in order to withstand with extreme process conditions.

To realise their full potential, enzymes undergo in-depth characterisation using tools from physical chemistry, structural biology and computational modelling. Multidisciplinary approaches combine these tools with genetic engineering, directed evolution or rational design to optimise enzyme activity, stability and specificity. Numerous studies are also aimed at improving immobilisation techniques and researching nanozymes—a new class of nanomaterials with characteristics similar to enzymes—which offer greater stability and lower costs for specific applications [7].

The Special Issue “The Characterisation and Application of Enzymes in Bioprocesses” in the *International Journal of Molecular Science* comprises two review articles and seven original research papers that provide significant insights into the field of enzymes and biocatalysts, as well as the progress made in their study and application.

In the first contribution, Salas-Garrucho et al. (Contribution 1) characterised a novel histidine ammonia-lyase (HAL) from the thermophilic bacterium *Geobacillus kaustophilus* (GkHAL), alongside eight active site mutants. HAL is an enzyme essential for the non-oxidative deamination of L-histidine to produce trans-urocanic acid, which plays a crucial role in amino acid metabolism. Mutational analysis has enabled the establishment of the structure–function relationship within GkHAL, identifying the residues essential for catalytic activity. These findings contribute to highlight their potential for industrial applications, particularly in environments requiring high thermal stability.

In their work, Monterrey et al. (Contribution 2) investigated *Burkholderia cenocepacia*, an opportunistic pathogen associated with cystic fibrosis, that exhibits elevated intracellular polyphosphate (polyP) accumulation, suggesting polyP metabolism as a target for novel biocatalysts. They characterised the polyphosphate kinase 2-III (BcPPK2-III) and the recombinant enzyme was structurally validated. Biochemical assays demonstrated that BcPPK2-III catalyses ATP synthesis from AMP using polyP. The enzyme exhibits broad divalent cation dependence, and kinetic analysis confirmed a stepwise sequential diphosphorylation mechanism, with ADP as an intermediate, also showing high thermo and pH stability. These characteristics make BcPPK2-III a promising candidate for ATP regeneration in biocatalytic processes, enabling cost-effective and scalable applications to be carried out under particularly challenging industrial conditions.

The development of improved cellulase-based bioprocesses, with enhanced enzyme production efficiency in industrial applications, was investigated in the original paper of Myeong et al. (Contribution 3). In this study, they optimised carbon source composition, agitation speed, and turbulence for enhanced cellulase production by *Trichoderma* sp. KMF006 in submerged fermentation (SmF), evaluating flask-scale parameters and bioreactor scale-up feasibility. The results provide a foundation for hydrodynamic optimisation in industrial SmF, highlighting the importance of balanced aeration, substrate accessibility and shear stress. A cellulolytic enzyme was also studied by Zuo et al. (Contribution 4), who enhanced the catalytic activity and stability of *Oenococcus oeni* β-glucosidase via site-directed mutagenesis targeting key residues in the catalytic pocket, yielding dominant mutants III and IV. Compared to the wild-type, mutants exhibited higher activity, improved thermal stability, and increased substrate affinity, with greater tolerance to low pH. Molecular docking revealed that hydrogen bonding and π–π interactions were the primary drivers of the enhanced binding of the enzyme to its substrate. These modifications expand the industrial applications of *O. oeni* β-glucosidase, particularly in enhancing the flavour of food and wine via glycoside hydrolysis, providing a foundation for enzyme engineering.

Gavrilova et al. (Contribution 5) explored the molecular functions of organelle-localised RNase H1. R-loops are three-stranded nucleic acid structures implicated in genome regulation/stabilisation and RNase H1 (RNH1) resolves R-loops and maintains genome stability in eukaryotic cells. RNH1C is essential for chloroplast development in *Arabidopsis thaliana* (AtRNH1C). The study characterised recombinant AtRNH1C in relation to R-loops of various structures using synthetic substrates, demonstrating that AtRNH1C cuts RNA within DNA/RNA hybrids, elucidating the molecular mechanism of a plant organelle-specific RNase H1. The work reveals the kinetic/mechanistic principles underlying the degradation of R-loops by RNase H1 enzymes, highlighting AtRNH1C’s specific adaptation to maintain homeostasis in the chloroplast environment. Another original paper by Bodakowska-Boczniewicz and Garncarek (Contribution 6) focuses on the characterisation of an enzyme complex. The study investigated the effect of immobilising naringinase on the optimal activity parameters of its subunits. Naringinase from *Aspergillus niger* KMS was adsorbed onto a carob gum support activated with polyethyleneimine. The two naringinase subunits, α-L-rhamnosidase and β-D-glucosidase, were characterised in various forms: free; immobilised on a magnetic polysaccharide carrier; and cross-linked with dextran aldehyde. The characterisation of the immobilisation of the two subunits is essential for their application, as it enables the targeted deglycosylation of flavonoids. This increases their bioavailability and antioxidant capacity, which are useful properties for food processing applications.

An interesting study by Tarara et al. (Contribution 7) demonstrates the biodegradation of UV-treated low-density polyethylene (LDPE) by a soil-derived bacterial consortium comprising *Pseudomonas* and *Bacillus* species. Over 30 days, the consortium achieved 7% mass loss for PE film and 13% for PE powder when these were used as the sole carbon sources. The analysis revealed surface oxidation and degradation products while metagenomic analysis identified an upregulation of a cytochrome P450 reductase from the *Bacillus thuringiensis* 9.1 strain, and the purified enzyme was found to modify the PE surface via oxidation. These findings elucidate the mechanisms by which the consortium synergistically degrades PE, highlighting bioremediation strategies for recalcitrant plastic waste. In the field of plastic waste degradation, the review by Ogunlusi et al. (Contribution 8) summarises the latest developments in the discovery, engineering and heterologous expression of *Ideonella sakaiensis* PETase and its homologues for polyethylene terephthalate (PET) biodegradation. This addresses the environmental crisis posed by persistent PET plastics. Key innovations include in silico/AI-driven enzyme screening, high-throughput assays and optimisation strategies for expression to boost secretion, solubility and yield across various microbial hosts. While laboratory successes demonstrate enhanced PET hydrolysis, persistent challenges include limitations of industrial-scale fermentation, stability of the enzymes under process conditions and integration into waste infrastructures. Future research will overcome these challenges in order to enable cost-effective and scalable PET recycling and bioremediation.

Leontakianakou et al. (Contribution 9) explored the potential of ferulic acid esterases (FAEs). These serine hydrolases act as debranching enzymes and cleave the ester bonds between ferulic acid and polysaccharides, facilitating biomass degradation via synergy with other CAZymes and enabling conversion of agricultural residues to fermentable sugars for biofuels and chemicals. The review provided an interesting and detailed account of the natural occurrence, forms and cross-linking mechanisms of FAEs, their classification systems and substrate specificities, their detection methods, and their versatile applications, including antioxidant, anti-inflammatory, anti-tumour and photoprotective properties in pharmaceutical and cosmetic products; use as vanillin precursors in food products; and synthesis of biopolymers. The biosynthetic potential of FAEs in non-aqueous (trans)esterification processes highlights opportunities for industrial exploitation and underscores the need for further advanced studies on the structure–function relationship.

This Special Issue features a diverse collection of articles that enhance our understanding of the potential of enzymatic biocatalysts across various sectors, including pharmaceuticals, food production, agriculture, environmental remediation, and bio-based manufacturing. Consequently, detailed characterisation and application of enzymes are essential to fully harness their potential as sustainable and versatile catalysts in modern bioprocesses.

## Figures and Tables

**Figure 1:**
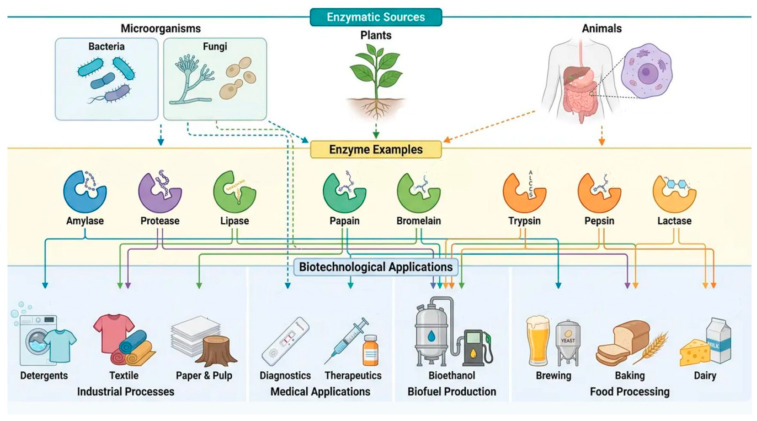
Examples of enzymatic sources and their potential applications in biotechnology. This figure was created with Motifolio (www.motifolio.com).
